# The Medical Faculty of London University

**Published:** 1913-04-26

**Authors:** 


### THE MEDICAL FACULTY OF LONDON UNIVERSITY.

Among the abstruse educational problems which the recent Royal Commission upon University Education in London was called upon to solve in its Report, few were more complicated than those connected with the medical side of the University's functions. The University of London is a thing of mushroom growth compared with many of the schools of medicine and surgery in London; and it had to adjust its regulations and its activities so as to interfere as little as possible with existing customs and systems of instruction, themselves evolved on no very definite plan. It thus happened that the medical degrees and curricula of the University presented many anomalies; and although on the whole it would be true to say that the University of London had raised the general standard of medical education, it was also the fact that in so doing it had incurred some unpopularity and had failed to spread its net wide enough. The University was prepared to hall-mark the highly instructed student, but took insufficient interest in the careers of those who did not reach the highest level of academic proficiency, though they might be in practice very able and dependable practitioners.

The Commissioners hold that the preliminary sciences of physics, chemistry, and biology should no longer be included in the University curriculum of medicine at all, but should appertain to the higher secondary schools. They would not admit a student to the faculty of medicine until the examinations in these subjects had been passed. With this bold solution we are in full agreement, provided that a high standard can be enforced, but such a desirable reform will hardly be achieved yet. The Commissioners report further that not only bacteriology and pathology, but also anatomy and physiology, are most advantageously taught in close proximity to the hospitals in which clinical teaching is given. This recommendation would seem at first sight to imply a condemnation of the system started a few years ago by a group of the smaller London schools of combining their forces for the teaching of preliminary and intermediate sciences. This plan possessed the merits of saving time, labour, and money, as well as promoting efficiency; and we think the Commissioners do not really disapprove of it, for they seem to contemplate the establishment of about three University medical colleges—attached to hospitals—in place of about double that number where the intermediate sciences are now taught. The importance of directing the study of anatomy and physiology along clinical lines is well exemplified by the innovation of a London University teacher a few weeks ago in showing to an anatomy class cases of paralysis which illustrated the distribution of various nerves to muscles.

In respect of clinical teaching the Commissioners admit many of the strong points of the London schools, and desire to see them preserved. But they would like to see University professors of the subjects of clinical instruction properly paid to teach, and permitted private practice only to a limited extent. Here we cannot pretend to agree with the Report. From the point of view of emoluments, the rewards of successful consulting practice are bound to be far greater than any professorial stipend. If, therefore, the professors are to be allowed any private practice at all, one of two things will happen. Either the best men will not consent to hold those posts, or the posts will be used as stepping stones to success in practice. If an attempt is to be made to get professors whose main business in life is to be teaching, it would be much better to forbid private practice altogether.

